# Synesthesia has specific cognitive processing during Go/No-go paradigms

**DOI:** 10.1038/s41598-023-32389-8

**Published:** 2023-04-15

**Authors:** Yu Aoki, Manabu Shibasaki, Hiroki Nakata

**Affiliations:** 1 Faculty of Human Life and Environment, Nara Women’s University, Nara, Japan; 2 Faculty of Engineering, Nara Women’s University, Kitauoya-Nishi Machi, Nara, 630-8506 Japan

**Keywords:** Neuroscience, Psychology

## Abstract

Grapheme-color synesthesia is a consistent and automatic perception of non-physical color when presented with a grapheme. Many previous studies focused on the synesthetic visual system, but other cognitive functions in grapheme-color synesthetes have remained unclear. Therefore, the objective of the present study was to investigate the characteristics of cognitive processing for motor execution and inhibition during Go/No-go paradigms in grapheme-color synesthesia using event-related potentials (ERPs). Six grapheme-color synesthetes and 24 non-synesthetes performed visual, auditory, and somatosensory Go/No-go paradigms. Omission errors were higher in grapheme-color synesthetes than non-synesthetes. Group-trial interactions (i.e., synesthetes–non-synesthetes × Go–No-go) were observed for the latency of the visual N2 component and amplitude of the somatosensory N2 component. Latencies of auditory and somatosensory P3 components were shorter in grapheme-color synesthetes than non-synesthetes. These findings suggest that grapheme-color synesthetes have specific cognitive processing in motor execution and inhibition as well as synesthetic color perception. Our data advance understanding of cognitive processing in grapheme-color synesthesia.

## Introduction

Synesthesia is the unusual perception in which a stimulus elicits two different and possibility conflicting (real and synesthetic) sensations at the same time. For example, grapheme-color synesthetes automatically perceive a particular color in association with a particular letter or digit^1^. Populations with synesthetes are very rare, but they are present in a certain proportion if grouped in certain ways. For example, Simner et al.^2^ reported 4.4% in 500 university students, and 1.1% in 1190 English speakers who visited London’s Science Museum.

Neuroanatomical studies of grapheme-color synesthesia using magnetic resonance imaging (MRI) showed larger grey matter in grapheme-color synesthetes than in non-synesthetes at the fusiform gyrus (FG) and posterior parietal cortex (PPC)^3^, and a globally hyperconnected brain architecture^1^. A functional MRI study also reported that neural activities in FG and PPC were related to grapheme-color synesthesia^4^. In addition, some studies using repetitive transcranial magnetic stimulation over the right PPC found transiently attenuated synesthesia^5,6^. These data suggest that PPC plays an important role in synesthetic color perception of grapheme-color synesthesia. However, beyond specific color perception in grapheme-color synesthetes, other higher cognitive functions such as inhibition and working memory have been unclear, because many studies focused on the synesthetic visual system.

Therefore, the objective of the present study was to investigate the characteristics of cognitive function during Go/No-go paradigms in grapheme-color synesthesia using event-related potentials (ERPs). Go/No-go paradigms have been used to investigate the cognitive function of motor execution and inhibition, and ERPs obtained by time-locked averaging electroencephalography (EEG) are used to clarify the temporal dynamics of cognitive processing during Go/No-go paradigms^7^. Two components, a negative deflection at approximately 140–300 ms (N2 component) after stimulus onset and a positive deflection at approximately 300–600 ms (P3 component), elicited in No-go trials were larger than the ERPs recorded in Go trials^8,9^. N2 and P3 components are elicited during visual, auditory, and somatosensory Go/No-go paradigms, respectively. N2 is generated from anterior midcingulate and inferior frontal sources, and the generator constellation underlying P3 covers precentral, middle frontal, and midcingulate areas^9^. Each component reflects different neural substrates of motor executive and inhibitive processing, which are clearly different from those of the synesthetic visual and linguistic systems.

In addition, some previous studies reported that PPC is one of the main and key generators of the P3 component^10,11^. PPC is related to cross-modal binding among visual, auditory, somatosensory, and spatial senses, and a sensorimotor interface^12,13^, as well as synesthetic visual perception. Thus, if grapheme-color synesthetes had a specific cognitive function and neural system in PPC, we hypothesized that the characteristics of the P3 component in ERPs would be different between grapheme-color synesthetes and non-synesthetes, even though they performed Go/No-go paradigms, independent of sensory modalities.

## Results

### Specific characteristics

**Figure 1:**
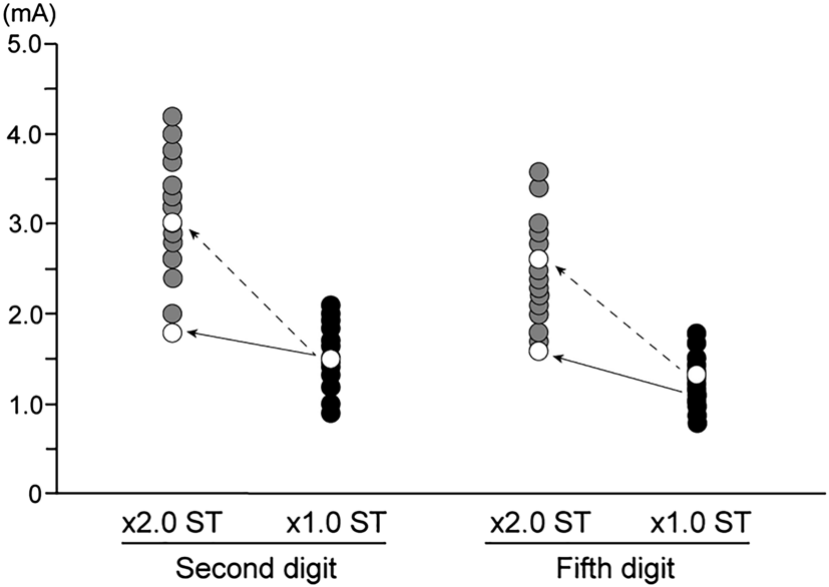
Stimulus intensity to second and fifth digits for sensory threshold and 2.0-times sensory threshold for synesthete B and 24 non-synesthetes. White circles indicate data on the stimulus intensity for synesthete B. Black circles show data on the stimulus intensity for 24 non-synesthetes. The sensory threshold (× 1.0 ST) for synesthete B is about the average of the 24 non-synesthetes. When set to twice the stimulus intensity, it is located at the value of the dotted arrow. However, synesthete B reported pain and an unpleasant sensation when the stimulus intensity was set to 2.0 times ST. Therefore, the stimulus intensity was set to 1.1 times ST, located at the value indicated by the arrow. *ST* sensory threshold.

Two synesthetes showed specific characteristics in behavioral and EEG data, while no such characteristic was observed from any non-synesthetes. Regarding background EEG activity, large waveforms such as alpha waves were periodically observed, and ERPs could not be measured in synesthete A (see movie for Supplementary Data 1). Therefore, this participant was excluded from ERP analysis. Synesthete B reported pain and an unpleasant sensation in somatosensory paradigms when the stimulus intensity was set even 2.0 times ST. Thus, in synesthete B, the stimulus intensity was set at 1.1 times ST, which caused no pain (Fig. 1).

### Behavioral data

Table 1 shows behavioral data during visual, auditory, and somatosensory Go/No-go paradigms in each synesthete. Table 2 shows behavioral data among synesthete and non-synesthete groups. Mann–Whitney tests showed a significant difference in visual paradigms for omission error and in auditory paradigms for commission error between groups (p < 0.01 and p < 0.05, respectively), indicating more errors in synesthetes than in non-synesthetes. Tukey HSD correction as a post-hoc test showed the significance for omission erroring in visual paradigms between groups. No significant differences between groups or interactions were observed in the reaction time (RT) or response variability (i.e., standard deviation (SD) of RT).

**Table 1:** Behavioral data during visual, auditory, and somatosensory Go/No-go paradigms in each synesthete.

	Sub A	Sub B	Sub C	Sub D	Sub E	Sub F	Ave
Score of Eagleman’s test	0.270	0.356	0.544	0.574	0.576	0.609	0.488 (0.140)
RT (ms)							
Visual	328	265	359	413	231	414	335 (76)
Auditory	323	224	295	393	198	482	319 (106)
Somatosensory	319	263	296	340	199	432	308 (78)
SD of RT (ms)							
Visual	60	50	56	64	35	80	58 (15)
Auditory	84	58	72	82	42	102	73 (21)
Somatosensory	101	67	82	96	45	102	82 (23)
Omission error (%)							
Visual	1.3	1.2	0	1.3	1.3	2.5	1.3 (0.8)
Auditory	3.8	0	1.2	3.8	0	7.5	2.7 (2.9)
Somatosensory	1.3	2.5	2.4	7.2	0	8.8	3.7 (3.5)
Commission error (%)							
Visual	0	2.5	1.3	0	7.4	0	1.9 (2.9)
Auditory	2.5	0	0	2.6	2.5	3.7	1.9 (1.5)
Somatosensory	1.3	1.2	0	0	2.5	2.4	1.2 (1.1)

**Table 2:** Behavioral data during visual, auditory, and somatosensory Go/No-go paradigms in synesthete and non-synesthete groups.

	Visual		Auditory		Somatosensory	
	Syn	Non-Syn	Syn	Non-Syn	Syn	Non-Syn
RT (ms)	335 (76)	316 (51)	319 (106)	306 (70)	308 (78)	309 (76)
SD of RT (ms)	57 (15)	50 (17)	73 (21)	66 (18)	82 (23)	70 (19)
Omission error (%)	1.2 (0.8)*	0.3 (0.6)	2.7 (2.9)	1.8 (2.0)	3.7 (3.5)	2.1 (2.7)
Commission error (%)	1.9 (2.9)	1.1 (1.6)	1.9 (1.5)*	0.6 (0.7)	1.2 (1.1)	0.9 (1.2)

Data are expressed as means (SDs). Syn = Synesthete; Non-syn = Non-synesthete. RT = reaction time. SD = standard deviation. Mann–Whitney test showed significant difference between groups as *p < 0.05.

### ERPs data

**Figure 2:**
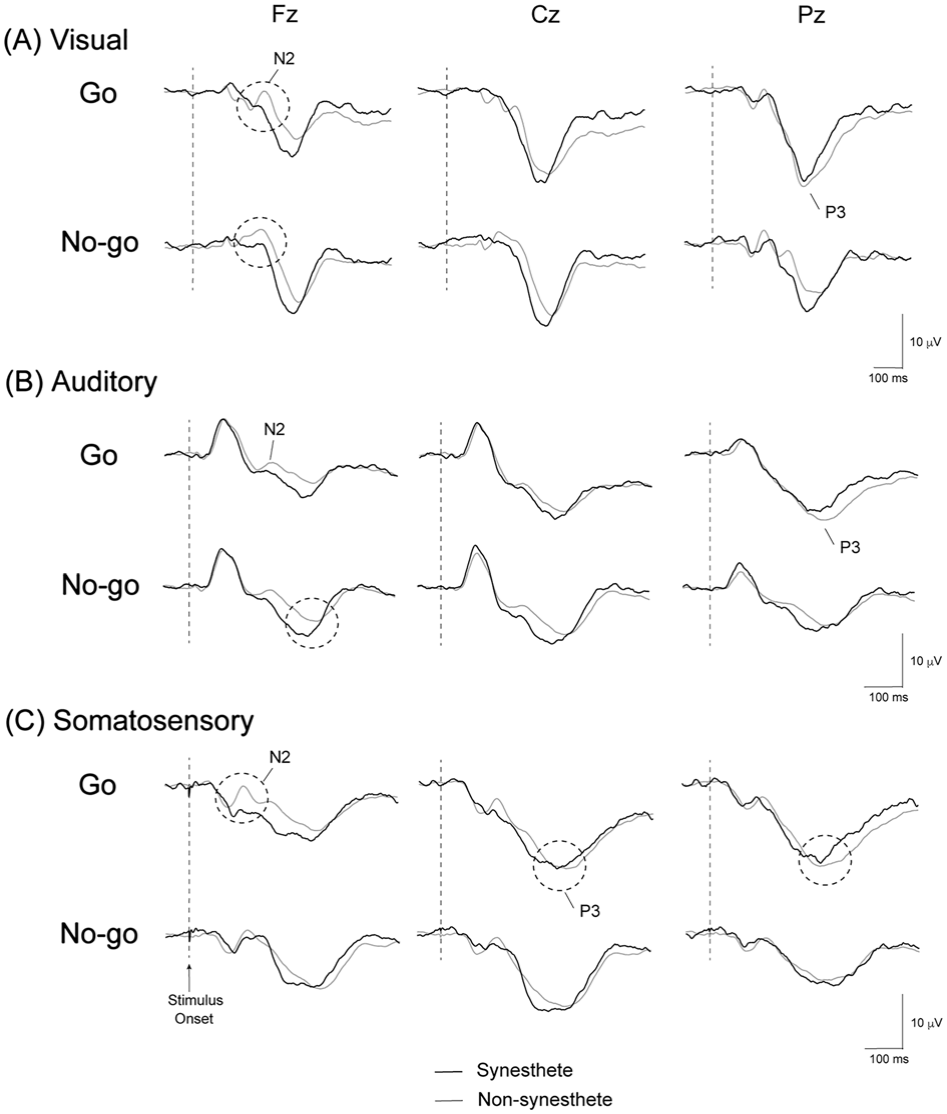
Grand-averaged ERP waveforms during (**A**) visual, (**B**) auditory, and (**C**) somatosensory Go/No-go paradigms. Black and grey lines indicate waveforms of synesthetes, and non-synesthetes, respectively.

Figure 2 shows grand-averaged waveforms of ERPs during visual, auditory, and somatosensory Go/No-go paradigms. The mean values for amplitudes and latencies of N2 and P3 components are listed in Tables 3 and 4, respectively.

**Table 3:** Mean values for latencies and amplitudes of N2 component in synesthete and non-synesthete groups with SD.

	Go	No-go	ANOVA		
			Main effect		interaction
			Group	Trial	Group-Trial
Latency (ms)					
Visual					
Syn	237 (8)	250 (8)	0.725	0.884	0.020
Non-syn	252 (21)	240 (20)			
Somatosensory					
Syn	142 (14)	161 (28)	0.865	0.025	0.064
Non-syn	152 (21)	154 (19)			
Amplitude (μV)					
Visual					
Syn	1.6 (3.0)	− 1.2 (2.6)	0.066	0.000	0.909
Non-syn	− 1.4 (3.4)	− 4.1 (3.6)			
Somatosensory					
Syn	2.0 (3.9)	− 1.8 (2.0)	0.232	0.000	0.021
Non-syn	− 0.9 (2.7)	− 1.8 (2.3)			

*Syn* Synesthete; *Non-syn* Non-synesthete.

Right rows indicate the results of ANOVAs.

**Table 4:** Mean values for the latency and amplitude of P3 component in synesthete and non-synesthete groups with SD.

	Go			No-go		
	Fz	Cz	Pz	Fz	Cz	Pz
Latency (ms)						
Visual						
Syn	363 (15)	345 (21)	330 (23)	374 (30)	383 (55)	345 (32)
Non-syn	365 (34)	359 (41)	336 (41)	383 (32)	381 (36)	364 (38)
Auditory						
Syn	305 (63)	294 (55)	312 (49)	293 (34)*	285 (34)*	306 (23)
Non-syn	329 (43)	320 (43)	314 (41)	327 (29)	324 (29)	321 (31)
Somatosensory						
Syn	293 (54)	266 (36)**	266 (18)*	302 (59)	300 (61)	308 (42)
Non-syn	328 (36)	324 (39)	312 (42)	330 (37)	316 (45)	315 (42)
Amplitude (μV)						
Visual						
Syn	11.8 (6.3)	15.0 (9.4)	15.5 (5.3)	11.9 (7.2)	13.6 (8.7)	11.3 (5.5)
Non-syn	9.9 (4.7)	16.7 (5.6)	18.7 (5.9)	11.0 (3.9)	13.5 (6.3)	10.6 (4.4)
Auditory						
Syn	7.2 (3.4)	10.2 (6.9)	10.4 (5.5)	8.3 (5.6)	9.6 (6.2)	8.0 (3.6)
Non-syn	6.2 (4.5)	11.9 (5.0)	13.5 (4.1)	7.3 (3.0)	10.0 (4.8)	8.3 (3.7)
Somatosensory						
Syn	10.1 (5.4)	14.7 (8.0)	13.7 (5.4)	11.1 (5.3)	14.6 (7.8)	9.4 (3.8)
Non-syn	9.3 (4.5)	16.6 (5.2)	16.1 (4.4)	11.4 (3.4)	15.2 (5.3)	10.5 (3.8)

ANOVA and Mann–Whitney test showed significant differences between groups as *p < 0.05 and **p < 0.01.

ANOVAs for the latency of visual N2 showed significant group-trial (i.e., synesthetes–non-synesthetes × Go–No-go) interaction (F (1, 27) = 6.092, p < 0.05). Further analysis showed that the latency of visual N2 was significantly longer in the Go trial than No-go trial among non-synesthetes (F (1, 23) = 7.924, p < 0.05), but not among synesthetes. ANOVAs for the peak amplitude of somatosensory N2 also showed significant group-trial interaction (F (1, 27) = 6.021, p < 0.05). Further analysis demonstrated that the amplitude of somatosensory N2 was significantly more negative in No-go trials than Go trials among synesthetes (F (1, 4) = 18.827, p < 0.05), but not among non-synesthetes (Table 3). Tukey HSD correction as a post-hoc test also showed the significance for the latency of visual N2 among non-synesthetes and for the amplitude of somatosensory N2 among synesthetes.

ANOVA showed that the peak latency of No-go-P3 during auditory paradigms was significantly shorter in synesthetes than in non-synesthetes at Fz (F (1, 27) = 5.534, p < 0.05) and Cz (F (1, 27) = 7.165, p < 0.05). ANOVA and the Mann–Whitney test demonstrated that the latency of Go-P3 during somatosensory paradigms was significantly shorter in synesthetes than in non-synesthetes at Cz (F (1, 27) = 9.075, p < 0.01) and Pz (p < 0.05). Tukey HSD correction as a post-hoc test showed the significance for the peak latency of No-go-P3 at Cz during auditory paradigms between groups, and for the latency of Go-P3 at Cz and Pz during somatosensory paradigms between groups. No significant differences in the peak latency of P3 during visual paradigms were observed between synesthetes and non-synesthetes. The results of ANOVAs for the amplitude of P3 showed no significant main effect of group, nor group-related interactions (Table 4).

ANOVAs for the latency of somatosensory N2 showed a strong tendency toward group-trial interaction (F (1, 27) = 3.716, p = 0.064). This interaction indicated that the latency of somatosensory Go-N2 was shorter among synesthetes than non-synesthetes, but that of No-go-N2 was longer among synesthetes than non-synesthetes. ANOVAs for the amplitude of visual N2 showed a strong tendency of group (F (1, 27) = 3.668, p = 0.066), suggesting a smaller amplitude in synesthetes than in non-synesthetes. Further analysis also demonstrated a strong tendency whereby the amplitude of somatosensory Go-N2 was smaller in synesthetes than non-synesthetes (F (1, 27) = 3.855, p = 0.060). These data showed strong tendencies, but were non-significant.

## Discussion

In the present study, we investigated the characteristics of cognitive function during Go/No-go paradigms in grapheme-color synesthesia using ERPs.

Two synesthetes showed specific characteristics in background EEG activity (synesthete A) and somatosensory threshold (synesthete B). Table 1 shows behavioral data on RT, the SD of RT, and omission and commission errors for each synesthete. Since the behavioral data of synesthete A were about the average, the arousal level would not affect the large waveforms. Figure 1 shows the stimulus intensity of ST and 2.0 times ST to second and fifth digits for synesthete B and 24 non-synesthetes. While the STs for synesthete B to both digits were about the average, 2.0-times ST could not be applied. These two subjects had the highest score (0.270) or the second highest score (0.356) in Eagleman’s test among all six synesthetes (Table 1). Thus, it is likely that participants with stronger synesthetic color perception have more specific neural networks than those with weak synesthetic perception.

Omission error rates in visual paradigms and commission error rates in auditory paradigms were significantly higher in grapheme-color synesthetes than in non-synesthetes, although other behavioral data were not different between the two groups. Sinke et al.^14^ also showed higher errors in synesthetes for response selection tasks with audio-visual simultaneous stimuli than in non-synesthetes. Since these errors reflect the number of failures to respond to a target stimulus and are related to inattentiveness^15^, we considered that the synesthetes are prone to inattention while performing cognitive tasks.

Two main findings were observed for ERP recording. One was group-trial interactions (i.e., synesthetes–non-synesthetes × Go–No-go) for the latency of visual N2 and the amplitude of somatosensory N2. The functional significance and precise origin of N2 have remained matters of debate. Previous studies suggested that N2 reflects motor inhibitory processing, and originates in the frontal lobe based on the topographical distribution^16,17^ or dipole modeling with ERP waveforms^18^. In contrast, other studies suggested that N2 reflects response conflict monitoring by the anterior cingulate cortex^19,20^. Based on these studies, frontal and/or conflict neural activities and these functions may be different between grapheme-color synesthetes and non-synesthetes.

The second was that the latency of P3 was shorter in grapheme-color synesthetes than in non-synesthetes, especially during somatosensory and auditory paradigms. The latency of P3 has been considered a measure of the stimulus classification and evaluation speed^21^, and is generally unrelated to response selection processes including RT^22^. This indicates that grapheme-color synesthetes have faster cognitive processing in motor executive and inhibitory processing, although no significant difference in RT was observed between two groups. We considered several possible explanations. The first hypothesis involves the specific neural networks. Several anatomical MRI studies reported unique networks of global hyperconnections^1^ and local regions^13^. Basically, these networks would be associated with eliciting synesthetic color, but other cognitive functions such as motor executive and inhibitory processing may also be affected. The second is hyperbinding in PPC. As mentioned in Introduction, PPC plays an important role in synesthetic color perception. In addition, generator mechanisms for P3 include PPC^10,11^, and PPC is related to cross-modal binding, spatial sense, information navigation and integration, and a sensorimotor interface^12,23^. Taking these into consideration, the specific neural activity and functional improvement in PPC among grapheme-color synesthetes might accelerate the latency of P3 during Go/No-go paradigms.

Moreover, the frontoparietal network may be related to the difference in latencies and/or amplitudes of N2 and P3 between grapheme-color synesthetes and non-synesthetes. The frontoparietal network is a control network, linking between frontal and parietal cortices, serving to rapidly and instantiate new task states by flexibly interacting with other control and processing networks^24^. Hupé and Dojat^25^ reported the strong connectivity in the frontoparietal network among grapheme-color synesthetes. Based on the generator mechanisms of N2 and P3, we considered that the shorter latencies of N2 and P3 among grapheme-color synesthetes were associated with the stronger connectivity of the frontoparietal network.

In the present study, all grapheme-color synesthetes were native Japanese speakers. Japanese use Latin alphabets and Arabic numerals as well as three types of Japanese script (Hiragana, Katakana, and Kanji) on a daily basis. Several studies already reported the characteristics of Japanese script in Japanese grapheme-color synesthetes^26,27^. Based on these, Japanese grapheme-color synesthetes might use different cognitive functions including the synesthetic visual and linguistic systems, and motor executive and inhibitive processing, compared with grapheme-color synesthetes in other countries only using Latin alphabets and Arabic numerals. If so, our findings might be specific to Japanese. This should be clarified in future studies.

As limitation of the present study, we could record only six female grapheme-color synesthetes, even though we recruited about 600 university students (i.e., 1%). Thus, larger numbers of and male grapheme-color synesthetes should be examined in future studies. Furthermore, other synesthetes, such as sound-color and olfactory-visual as well as grapheme-color, should be studied, because cognitive specification and neural networks of synesthesia might differ. We focused on the peak amplitudes and latencies of N2 and P3 components at five electrodes, which were sufficient to show the difference in brain potentials between grapheme-color synesthetes and non-synesthetes. However, the present study could not directly address which brain regions were different because we did not use dipole or independent component analyses involving a multi-channel EEG system.

In conclusion, the present study investigated cognitive processing during Go/No-go paradigms in grapheme-color synesthesia using behavioral data and ERPs. We found that grapheme-color synesthetes exhibit specific cognitive processing in motor execution and inhibition as well as synesthetic color perception. Our data provide findings to advance understanding of cognitive processing in grapheme-color synesthesia.

## Methods

### Ethical approval

Informed consent was obtained from all participants. This study was approved by the Ethical Committee of Nara Women’s University, Nara City, Japan (18-21). Experimental procedures and the protocol conformed to the Declaration of Helsinki.

### Subjects

Six female grapheme-color synesthetes (mean age: 20.2 ± 1.3 years) and 24 female non-synesthete controls (mean age: 21.3 ± 0.8 years) participated in the study. Non-synesthetes were screened with a detailed questionnaire to ensure that they did not experience synesthesia. None of the participants reported a history of neurological disorders. All participants were native Japanese speakers.

To recruit synesthetes, a preliminary questionnaire was conducted involving about 600 university students to see if they had grapheme-color synesthesia, based upon a previous study^2^. All the synesthetes reported experiencing synesthetic colors when viewing Japanese Hiragana, Katakana, and Kanji characters as well as Latin alphabets and Arabic numerals. The synesthetes participated in two experiments. The first employed Eagleman’s test, which was a color-selection task to determine the synesthetic colors^28^. Participants were presented with randomly ordered graphemes a total of three times each (108 trials). Using a color palette, participants chose the color that best matched their synesthetic percept for that grapheme. The test was conducted in a room with the lights turned off to avoid color deterioration. As for the scoring, a perfect score of 0.0 for the color match consistency test means that there is no difference in the colors selected on each successive presentation of the same letter. A score of 1.0 is defined as the threshold for synesthetic classification^28^. Scores below 1.0 indicate higher color match consistency^29^. The average score among the six synesthetes was 0.488 ± 0.112 (Table 1).

### Experimental procedure

On the second day, all participants performed visual, auditory, and somatosensory auditory Go/No-go paradigms. The order of the three paradigms was randomized for each participant and counterbalanced across all participants. In the visual Go/No-go paradigms, visual stimuli were presented on a TV monitor approximately 1 m in front of the subjects using a personal computer programmed by the authors (Hewlett-Packard xw4400 Workstation). Go and No-go stimuli were green and red circles (500-ms duration), respectively, presented in the center of the monitor. The background was black. In the auditory Go/No-go paradigm, auditory stimuli were presented binaurally through headphones (65-dB sound pressure level, 500-ms duration). Go and No-go stimuli were pure tones of 1500 and 1000 Hz, respectively. In the somatosensory Go/No-go paradigm, the Go stimulus was delivered to the second digit of the left hand, and the No-go stimulus to the fifth digit of the left hand with ring electrodes. The electrical stimulus used was a current constant square wave pulse of 0.2 ms in duration. The stimulus intensity was 2 times, a sensory threshold (ST) that yielded no pain. The interstimulus interval was 2 s. The probability of Go and No-go stimuli was the same in random order.

Participants were instructed to keep their eyes open. They had to respond by pushing a button with their right thumb as quickly as possible only after the presentation of a Go stimulus. One run comprised 80 epochs of stimulation, which included 40 epochs for the Go stimuli and 40 for the No-go stimuli, and two runs were performed for the visual, auditory, and somatosensory paradigms, respectively (i.e., six runs in total). The order of conditions was randomized for each subject and counterbalanced across all subjects. In a practice run, subjects were instructed to perform the task for 10 stimuli before recording.

### EEG recordings

EEG was recorded at Fz, Cz, Pz, C3, and C4. Each scalp electrode was referenced to linked earlobes. In order to exclude eye movements or blinks exceeding 100 μV, an electrooculogram was recorded bipolarly with a pair of electrodes placed 2 cm lateral to the lateral canthus of the left eye and 2 cm above the upper edge of the left orbit and analyzed on-line. Impedance was maintained at less than 5 kohm. All EEG signals were collected on a signal processor (Neuropack MEB-2300 system, Nihon-Kohden, Tokyo, Japan). The peak amplitudes and latencies of N2 components for the visual, auditory, and somatosensory paradigms were measured at 180–280, 180–230, and 110–210 ms, respectively, while those of P3 components were 280, 260–460, and 230–430 ms, respectively. Each amplitude was calculated by the baseline-to-peak. Slow responses exceeding 700 ms and incorrect responses were eliminated from averaging. As behavioral data, RT, the SD of RT, and omission and commission errors were evaluated for each condition.

### Data analysis

We checked for the normal distribution of behavioral and ERP data using the Kolmogorov–Smirnov test. If normal distributions were observed, the data were subjected to analysis of variance (ANOVA) to compare the difference between groups (synesthetes vs. non-synesthetes). If non-normal data distributions were observed, the data underwent Mann–Whitney tests. The amplitudes and latencies of visual and somatosensory N2 components at Fz were separately analyzed by ANOVA using factors of group and trial (Go vs. No-go). The auditory N2 components were not recorded from all participants. Thus, we deleted them from analysis. The amplitude of the P3 component was submitted to ANOVA using group, modality, trial, and electrode (Fz, Cz, and Pz). As for the latency of the P3 component, since non-normal data distributions were observed at some electrodes, ANOVA and Mann–Whitney tests were separately performed to compare the data between group at each electrode. Significance was set at p < 0.05.

### Ethics approval and consent to participate

All procedures performed in studies involving human participants were in accordance with the ethical standards of the institutional and/or national research committee and with the 1964 Helsinki declaration and its later amendments or comparable ethical standards. All the participants signed the informed consent form agreeing to submit to the procedures involved in the study.

## Supplementary Information

Supplementary Information.

## Data Availability

All relevant data are available from the corresponding author on reasonable request.

## Abbreviations

ERPs: Event-related potentials
MRI: Magnetic resonance imaging
FG: Fusiform gyrus
PPC: Posterior parietal cortex
EEG: Electroencephalography
ST: Sensory threshold
RT: Reaction time
