# Supplementary material for: Synesthesia has specific cognitive processing during Go/No-go paradigms
Source: Sci Rep. 2023 Apr 15;13:6172. doi: 10.1038/s41598-023-32389-8 (PMC10105738; doi:10.1038/s41598-023-32389-8)
Supplement: Supplementary file 1 — Supplementary Information. [file 41598_2023_32389_MOESM1_ESM.docx]

Supplementary data

Data 1: Background EEG activities of synesthetes and non-Synesthetes.

https://youtu.be/fEkYtD95XU8

https://youtu.be/tD-kLqGM7qw
